# Supplementary material for: Financial constraints and corporate bankruptcy risks in China: The buffer role of cash holdings
Source: PLoS One. 2026 Jan 27;21(1):e0341114. doi: 10.1371/journal.pone.0341114 (PMC12843545; doi:10.1371/journal.pone.0341114)
Supplement: S1 Appendix — (DOCX) [file pone.0341114.s001.docx]

**Appendix A: Variable definitions**

| **Variable** | **Notation** | **Descriptions** |
| --- | --- | --- |
| **Dependent variables** |  |  |
| Z-score | Z-SCORE | We follow Altman [1] to estimate the Z-score as the following formula:  Z-score = 1.2a + 1.4b + 3.3c + 0.6d + 1.0e  Where:  a: Working capital / Total assets.  b: Retained Earnings / Total assets.  c: Earnings before interest and taxes / Total assets.  d: Market value of equity / Book value of total debt.  e: Sales / Total assets. |
| ZM-score | ZM-SCORE | We follow Zmijewski [14] and Boubaker et al. [39] to estimate the ZM-score as the following formula:  ZM-score = -4.336 – 4.513a + 5.679b + 0.004c  Where:  a: Net income / Total assets.  b: Total liabilities / Total assets.  c: Current assets / Current liabilities. |
| Z’-score | Z’-SCORE | We follow Altman [13] to estimate the Z’-score as the following formula:  Z-score = 6.56a + 3.26b + 6.72c + 1.05d  Where:  a: Working capital / Total assets.  b: Retained Earnings / Total assets.  c: Operating income / Total assets.  d: Book value of equity /Total liabilities. |
| Z”-score | Z”-SCORE | We follow Altman [13] to estimate the Z”-score (for emerging markets) as the following formula:  Z-score = 3.25 + 6.56a + 3.26b + 6.72c + 1.05d  Where:  a: Working capital / Total assets.  b: Retained Earnings / Total assets.  c: Operating income / Total assets.  d: Book value of equity /Total liabilities. |
| **Independent variables** |  |  |
| Financial Constraints | SA | SA index represents financial constraints [6,7] |
| Cash Holdings | CASH | Total cash and short-term investments divided by total book assets [31] |
| **Control variables** |  |  |
| Fixed Asset Turnover | FAT | The ratio of net sales to average fixed asset [40] |
| Fixed Assets To Total Assets | FTA | The ratio of fixed assets to total assets [41] |
| Net Income Growth | NIG | The growth of net income [42] |
| Net Profit Margin | NPM | The ratio of net profit to net sales [43] |
| Total asset growth | TAG | The growth of total assets [44] |
| Return on assets | ROA | The net income is divided by the average total assets [3] |
