# Supplementary material for: Financial constraints and corporate bankruptcy risks in China: The buffer role of cash holdings
Source: PLoS One. 2026 Jan 27;21(1):e0341114. doi: 10.1371/journal.pone.0341114 (PMC12843545; doi:10.1371/journal.pone.0341114)
Supplement: Attachment — Submitted filename: Response-to-reviewer-report.edited.docx [file pone.0341114.s003.docx]

**RESPONSES TO REVIEWER'S REPORT**

PONE-D-24-39978

FINANCIAL CONSTRAINTS AND CORPORATE BANKRUPTCY RISKS IN CHINA: THE BUFFER ROLE OF CASH HOLDINGS

PLOS ONE

Dear editorial office and anonymous reviewers,

Thank you for allowing us to revise and resubmit our paper to your PLOS ONE. Thank you for your time and efforts in reviewing our paper. Thank you and the reviewers for giving us excellent comments, which helped us significantly improve our manuscript.

Below are our responses to helpful comments and suggestions.

Journal Requirements:

When submitting your revision, we need you to address these additional requirements.

1. Please ensure that your manuscript meets PLOS ONE's style requirements, including those for file naming. The PLOS ONE style templates can be found at

https://journals.plos.org/plosone/s/file?id=wjVg/PLOSOne_formatting_sample_main_body.pdf and

https://journals.plos.org/plosone/s/file?id=ba62/PLOSOne_formatting_sample_title_authors_affiliations.pdf

2. We note that the grant information you provided in the ‘Funding Information’ and ‘Financial Disclosure’ sections do not match.

When you resubmit, please ensure that you provide the correct grant numbers for the awards you received for your study in the ‘Funding Information’ section.

3. Thank you for stating the following financial disclosure:

“This study is supported by Ton Duc Thang University. None of the authors receive specific funding numbers for this study.”

Please state what role the funders took in the study. If the funders had no role, please state: "The funders had no role in study design, data collection and analysis, decision to publish, or preparation of the manuscript." If this statement is not correct you must amend it as needed.

Please include this amended Role of Funder statement in your cover letter; we will change the online submission form on your behalf.

4. We note that you have indicated that there are restrictions to data sharing for this study. PLOS only allows data to be available upon request if there are legal or ethical restrictions on sharing data publicly. For more information on unacceptable data access restrictions, please see http://journals.plos.org/plosone/s/data-availability#loc-unacceptable-data-access-restrictions.

Before we proceed with your manuscript, please address the following prompts:

a) If there are ethical or legal restrictions on sharing a de-identified data set, please explain them in detail (e.g., data contain potentially identifying or sensitive patient information, data are owned by a third-party organization, etc.) and who has imposed them (e.g., a Research Ethics Committee or Institutional Review Board, etc.). Please also provide contact information for a data access committee, ethics committee, or other institutional body to which data requests may be sent.

b) If there are no restrictions, please upload the minimal anonymized data set necessary to replicate your study findings to a stable, public repository and provide us with the relevant URLs, DOIs, or accession numbers. For a list of recommended repositories, please see

https://journals.plos.org/plosone/s/recommended-repositories. You also have the option of uploading the data as Supporting Information files, but we would recommend depositing data directly to a data repository if possible.

We will update your Data Availability statement on your behalf to reflect the information you provide.

5. When completing the data availability statement of the submission form, you indicated that you will make your data available on acceptance. We strongly recommend all authors decide on a data sharing plan before acceptance, as the process can be lengthy and hold up publication timelines. Please note that, though access restrictions are acceptable now, your entire data will need to be made freely accessible if your manuscript is accepted for publication. This policy applies to all data except where public deposition would breach compliance with the protocol approved by your research ethics board. If you are unable to adhere to our open data policy, please kindly revise your statement to explain your reasoning and we will seek the editor's input on an exemption. Please be assured that, once you have provided your new statement, the assessment of your exemption will not hold up the peer review process.

6. Please include captions for your Supporting Information files at the end of your manuscript, and update any in-text citations to match accordingly. Please see our Supporting Information guidelines for more information: http://journals.plos.org/plosone/s/supporting-information.

[Note: HTML markup is below. Please do not edit.]

Reviewers' comments:

Reviewer's Responses to Questions

**Comments to the Author**

1. Is the manuscript technically sound, and do the data support the conclusions?

The manuscript must describe a technically sound piece of scientific research with data that supports the conclusions. Experiments must have been conducted rigorously, with appropriate controls, replication, and sample sizes. The conclusions must be drawn appropriately based on the data presented.

Reviewer #1: Partly

Reviewer #2: Partly

2. Has the statistical analysis been performed appropriately and rigorously?

Reviewer #1: Yes

Reviewer #2: Yes

3. Have the authors made all data underlying the findings in their manuscript fully available?

The [PLOS Data policy](http://www.plosone.org/static/policies.action#sharing) requires authors to make all data underlying the findings described in their manuscript fully available without restriction, with rare exception (please refer to the Data Availability Statement in the manuscript PDF file). The data should be provided as part of the manuscript or its supporting information, or deposited to a public repository. For example, in addition to summary statistics, the data points behind means, medians and variance measures should be available. If there are restrictions on publicly sharing data—e.g. participant privacy or use of data from a third party—those must be specified.

Reviewer #1: No

Reviewer #2: Yes

4. Is the manuscript presented in an intelligible fashion and written in standard English?

PLOS ONE does not copyedit accepted manuscripts, so the language in submitted articles must be clear, correct, and unambiguous. Any typographical or grammatical errors should be corrected at revision, so please note any specific errors here.

Reviewer #1: Yes

Reviewer #2: Yes

5. Review Comments to the Author

Please use the space provided to explain your answers to the questions above. You may also include additional comments for the author, including concerns about dual publication, research ethics, or publication ethics. (Please upload your review as an attachment if it exceeds 20,000 characters)

Reviewer #1: The authors provide a worthy contribution to the dense discussion on the impact of cash holdings and financial constraints on firms' bankruptcy risks. Specifically, they focus on 25,002 firm-year observations in the period of 2010-2023, examining only publicly listed A-share companies on the **Shanghai** and **Shenzen** stock exchanges. The findings they present align with the expectations of both the market timing theory and the trade-off theory - large financially constrained firms with large cash holdings exhibit lower bankruptcy risk with their Z-score (the primary measure of risk in the paper) rising by ~2.2 points (going by the GMM estimations). The paper is well-structured, concise and tries to lend scrutiny to the authors' findings. However, certain parts of the paper could use improvement.

1. Zhang et al. [9] provides an analysis similar to yours, but only for the Shanghai stock exchange. Your results corroborate their findings, however I would also be interested if there are any differences in the effects you find across the Shanghai and Shenzen stock exchanges. Is the "listing location" something that is available to you? Ideally, you can include a firm's location proxy in the regressions as an **additional fixed effect**, or you can create heterogeneous effects based on these.

**Our responses**: We sincerely appreciate the insightful suggestions. We have examined subsamples from the Shanghai and Shenzhen stock exchanges; however, our findings do not indicate statistically significant differences in the effects. The results are presented in Table 8 below.

2. As a robustness measure you introduce the adjusted AWW index along with the KZ index which are both proxies for financial constraint. However, you omit the AWW from table 2 and table 6. What is the reasoning behind this?

**Our responses**: We thank anonymous reviewers for their valuable feedback. Initially, we considered AKZ and AWW indexes in our primary analysis but decided to focus on AKZ as the primary proxy while using AWW in the robustness test. Since different proxies capture varying aspects of financial constraints, incorporating AWW in the robustness test helps verify our findings' consistency and strengthens our conclusions' reliability. The revised section is Pages 18-20, highlighted in the manuscript.

3. You note: "We also follow Le et al. [24] to winsorize all variables at the 5% and 95% levels to minimize extreme values bias". I consider a winsor that applies to 10% of the observations you use to be at the very least unrepresentative by itself, regardless of the paper you refer to, or the amount of the data at your disposal. Winsoring the data is of course permitted, but I would advise against going over the 2.5% border on either side. Also, you maintain a great deal of the observations you start with even with the usual lag observation losses in GMM - this allows you to trim your sample and avoid any potential confusion. I would wish to see:

• the regression results without any winsoring/trimming,

• the results when winsoring the data on the top and bottom 1% symmetrically, and

• the results when trimming the observations the same way.

Ideally, one of these should be the main paper estimates, or you should provide a more sufficient reason why you winsor on a total of 10% of observations.

**Our response:** We appreciate the valuable suggestion. To address this concern, we have conducted regressions without winsorizing or trimming and have chosen these results as our main estimates. This approach ensures that our findings are not influenced by data adjustments while maintaining the robustness of our analysis.

4. You split the firms into the manufacturing and non-manufacturing industries, you use the ISG (industry sales growth) in your financial constraint index creation, and you use the industry fixed effects in the regressions. However, you do not note what industry variable you base this on? Is this always a 2-digit sector, as in **Chan et al. [5] you reference**, or is this something else? This should be made clear and consistent throughout the paper.

**Our response:** We thank anonymous reviewers for their valuable feedback. In our previous manuscript, the sector classification used in our analysis is consistent with the two-digit sector classification referenced in Chan et al. [5].

5. Another point regarding your split on the manufacturing and non-manufacturing groups of firms. It is true that your results align with the Altman's paper, however your observation count in table 6 is rather odd. **18,367 vs. 1,729** observations seems a quite unbalanced comparison. How do you actually split the firms into these two groups, and is there any way you could improve this split (in terms of observation count balance) with the data available to you?

**Our response:** We thank anonymous reviewers for their valuable feedback. Following the reviewer's feedback, we realized that the difference between the two industry-based subsamples was too substantial. Therefore, we decided not to divide the firms by industry in the revised analysis. Instead, we follow Fama and French (1993) to split the data into two subsamples based on firm size. The revised section is in Table 7, highlighted in the manuscript.

6. My last major point is your conclusion. Throughout your paper you note that (based on the literature so far) SME's and large firms differ in the effects financial constraints and cash holdings have on the bankruptcy risk of said firms. I assume, since you have not provided any **non-ratio descriptive statistics on the firms in your sample**, that A-share listed companies are by and large non-SME's. However, you conclude with the practical implications that pertain exactly to SMEs. I would be wary of such interpretations without any "raw" descriptive statistics nor SME-specific heterogeneous effects on the firms in your sample. You should provide the readers with more context on the firms you work with, at the very least for all the starting variables you use in your analysis before any ratios.

**Our response:** We sincerely appreciate valuable and insightful comments that have helped us improve the quality of our manuscript. Following Fama and French (1993), we classify firms by size: small (below 30th percentile), medium (30th–40th), and large (above 40th). We then group small and medium firms as SMEs to analyze their distinct effects, ensuring our conclusions align with the sample context.

Minor notes:

• You write the 3.4. section as if it was done in advance. In my opinion it makes it only harder to read and understand, with little benefit to it.

**Our responses**: We thank anonymous reviewers for their valuable feedback. We have revised Section 3.4 to improve clarity and ensure the methodology is more structured and readable. The updated section is on Page 13, highlighted in the manuscript.

• You have two H2 hypotheses, I don't know if this is intentional or not, but I suggest you change it to H2 and H3.

**Our responses:** We thank anonymous reviewers for their valuable feedback. This issue was unintentional, and I have corrected it by renaming the second H2 to H3 for clarity and consistency. The revised hypotheses are on Page 8 and Page 10, highlighted in the manuscript.

• "We select the sampling period is from 2010 to prevents tha adverse impacts of the financial crisis in 2008 on the findings." - typo on pg. 3 and pg. 8.

**Our responses:** We thank an anonymous reviewer for identifying this typo. We have corrected it to ensure clarity and accuracy in the text. The updated sentences are on Pages 3 and 11, highlighted in the manuscript.

Reviewer #2: The introduction section is poorly managed. It is likely a literature review. It requires rewriting: The introduction section of a typical research is, needless to say, like a driving force that leads to the rest of the paper and thus it should be carefully written. The introduction should properly present (in no particular order but in a logical manner) the background information, the motivation of the study, the research questions, the gaps in the literature that the study fills, contribution of the study and so on. It is suggested to rewrite the introduction in order to make it sufficient, better sequenced and having read smoothly for the readers.

**Our responses:** Thank you for your detailed feedback. We have restructured and rewritten the introduction to ensure a more precise flow. The revised section is on Pages 2-5, highlighted in the manuscript.

The authors have discussed two alternative theories supporting the relationship between cash holdings and bankruptcy risk (hypothesis 1). However, they propose a single relationship in the hypothesis. I think that it is better to propose two alternative hypotheses relating to each theory. The same case for hypothesis 2.

**Our responses:** We thank anonymous reviewers for their valuable feedback. We have revised the hypotheses to reflect the alternative theories by splitting them into H1A, H1B, H2A, and H2B for better clarity and alignment with the theoretical framework. The revised hypotheses are on Page 8 and Page 10, highlighted in the manuscript.

The author(s) need to link their findings more strongly to context, highlight their economic, academic/research, and policy/practice implications.

**Our responses:** We thank anonymous reviewers for their valuable feedback. We have revised the conclusion section to highlight our findings' economic, academic, and policy implications. The updated section is on Page 25, highlighted in the manuscript.

6. PLOS authors have the option to publish the peer review history of their article ([what does this mean?](https://journals.plos.org/plosone/s/editorial-and-peer-review-process#loc-peer-review-history)). If published, this will include your full peer review and any attached files.

If you choose "no", your identity will remain anonymous but your review may still be made public.

**Do you want your identity to be public for this peer review?** For information about this choice, including consent withdrawal, please see our [Privacy Policy](https://www.plos.org/privacy-policy).

Reviewer #1: No

Reviewer #2: **Yes:** Moncef Guizani

[NOTE: If reviewer comments were submitted as an attachment file, they will be attached to this email and accessible via the submission site. Please log into your account, locate the manuscript record, and check for the action link "View Attachments". If this link does not appear, there are no attachment files.]

While revising your submission, please upload your figure files to the Preflight Analysis and Conversion Engine (PACE) digital diagnostic tool, <https://pacev2.apexcovantage.com/>. PACE helps ensure that figures meet PLOS requirements. To use PACE, you must first register as a user. Registration is free. Then, login and navigate to the UPLOAD tab, where you will find detailed instructions on how to use the tool. If you encounter any issues or have any questions when using PACE, please email PLOS at [figures@plos.org](mailto:figures@plos.org). Please note that Supporting Information files do not need this step.

*In compliance with data protection regulations, you may request that we remove your personal registration details at any time.* [*(Remove my information/details)*](https://www.editorialmanager.com/pone/login.asp?a=r)*. Please contact the publication office if you have any questions.*

**References**

5.	Chan H, Chang X, Faff R, Wong G. Financial constraints and stock returns — Evidence from Australia. Pacific-Basin Finance Journal. 2010;18: 306–318. doi:10.1016/j.pacfin.2010.02.004

12.	Fama EF, French KR. Common risk factors in the returns on stocks and bonds. J financ econ. 1993;33: 3–56. doi:10.1016/0304-405X(93)90023-5

**Table 8: Robustness test by employing Shanghai and Shangzhen stock exchanges.**

| **VARIABLES** | **Shanghai** | **SHANGZHEN** |
| --- | --- | --- |
| **Zscore (-1)** | 0.2238*** | 0.059*** |
|  | (<0.001) | (<0.001) |
| **AKZ** | -0.0105 | -4.621*** |
|  | (0.196) | (<0.001) |
| **CASH** | -1.5195 | -11.688*** |
|  | (0.511) | (<0.001) |
| **AKZCASH** | 0.1001** | 17.499*** |
|  | (0.042) | (<0.001) |
| **AGE** | -11.7822*** | -860.505*** |
|  | (<0.001) | (<0.001) |
| **LEV** | 1.7538*** | 2.378*** |
|  | (<0.001) | (<0.001) |
| **FTA** | 1.0460 | 23.796** |
|  | (0.791) | (0.017) |
| **NSG** | -0.1521*** | -0.002 |
|  | (<0.001) | (0.612) |
| **ROA** | 15.8661*** | 9.547*** |
|  | (<0.001) | (<0.001) |
| **Cross-Section Fixed** | Yes | Yes |
| **Period Fixed** | Yes | Yes |
| **J-Statistic** | 117.2877 | 104.9874 |
| **Prob(J-Statistic)** | 0.0003 | 0.0034 |
| **Instrument Rank** | 90 | 90 |
| **N** | 14,674 | 23,790 |

*Notes: Table 8 presents the robustness results from GMM estimations of different stock exchange samples. All variable definitions are reported in Appendix A. ∗, ∗∗, ∗∗∗ indicating significance at 10%, 5%, and 1%, respectively. P-values are in parentheses.*
