# Supplementary material for: Financial constraints and corporate bankruptcy risks in China: The buffer role of cash holdings
Source: PLoS One. 2026 Jan 27;21(1):e0341114. doi: 10.1371/journal.pone.0341114 (PMC12843545; doi:10.1371/journal.pone.0341114)
Supplement: Attachment — Submitted filename: Response to Reviewer.docx [file pone.0341114.s004.docx]

PONE-D-24-39978R1

FINANCIAL CONSTRAINTS AND CORPORATE BANKRUPTCY RISKS IN CHINA: THE BUFFER ROLE OF CASH HOLDINGS

PLOS ONE

Journal Requirements:

Please review your reference list to ensure that it is complete and correct. If you have cited papers that have been retracted, please include the rationale for doing so in the manuscript text, or remove these references and replace them with relevant current references. Any changes to the reference list should be mentioned in the rebuttal letter that accompanies your revised manuscript. If you need to cite a retracted article, indicate the article’s retracted status in the References list and also include a citation and full reference for the retraction notice.

**Comments to the Author**

1. If the authors have adequately addressed your comments raised in a previous round of review and you feel that this manuscript is now acceptable for publication, you may indicate that here to bypass the “Comments to the Author” section, enter your conflict of interest statement in the “Confidential to Editor” section, and submit your "Accept" recommendation.

Reviewer #1: (No Response)

Reviewer #3: (No Response)

2. Is the manuscript technically sound, and do the data support the conclusions?

The manuscript must describe a technically sound piece of scientific research with data that supports the conclusions. Experiments must have been conducted rigorously, with appropriate controls, replication, and sample sizes. The conclusions must be drawn appropriately based on the data presented.

Reviewer #1: Partly

Reviewer #3: Yes

3. Has the statistical analysis been performed appropriately and rigorously?

Reviewer #1: Yes

Reviewer #3: Yes

4. Have the authors made all data underlying the findings in their manuscript fully available?

The [PLOS Data policy](http://www.plosone.org/static/policies.action#sharing) requires authors to make all data underlying the findings described in their manuscript fully available without restriction, with rare exception (please refer to the Data Availability Statement in the manuscript PDF file). The data should be provided as part of the manuscript or its supporting information, or deposited to a public repository. For example, in addition to summary statistics, the data points behind means, medians and variance measures should be available. If there are restrictions on publicly sharing data—e.g. participant privacy or use of data from a third party—those must be specified.

Reviewer #1: No

Reviewer #3: Yes

5. Is the manuscript presented in an intelligible fashion and written in standard English?

PLOS ONE does not copyedit accepted manuscripts, so the language in submitted articles must be clear, correct, and unambiguous. Any typographical or grammatical errors should be corrected at revision, so please note any specific errors here.

Reviewer #1: Yes

Reviewer #3: Yes

6. Review Comments to the Author

Please use the space provided to explain your answers to the questions above. You may also include additional comments for the author, including concerns about dual publication, research ethics, or publication ethics. (Please upload your review as an attachment if it exceeds 20,000 characters)

**Reviewer #1**: The authors have, in my opinion, made reasonably sufficient improvements in response to my previous comments. However, I still have a few hanging questions. My comments here follow the same order (numbers) as the previous review.

1. Thank you for addressing my suggestion on the location split and for conducting the subsample analysis. Given that the results do not indicate statistically significant differences, I recommend moving them to the appendix rather than including them in the main findings.

**Our responses:** We thank anonymous reviewers for their valuable feedback. Following the reviewer's feedback, we have moved Table 8 to Appendix B.

2. I respect your choice not to include the AWW in your main results. However, since AWW is still used to validate the robustness of your findings, I recommend including it in the Table 2 correlation matrix to provide a more comprehensive overview of the relationships among the key variables, as AWW is one too.

**Our responses**: We sincerely appreciate the insightful suggestions. Following the reviewer's feedback, we have added AWW into the Table 2 correlation matrix. The revised section is in Table 2, highlighted in the manuscript.

5. Thank you for reconsidering the industry-based split and for adopting the Fama and French (1993) approach based on firm size. This is clearly a more suitable alternative in your case. However, I would advise against labeling these firms as SMEs, as the SME classification is often based on the EU definition, which differs from this approach. This clarification would help avoid potential misinterpretations.

**Our responses**: We sincerely appreciate the insightful suggestions. Following the reviewer's feedback, we have changed "SMEs" to "Small and Medium-sized firms" to avoid potential misinterpretations.

6. Again, incorporating the size-based classification following Fama and French (1993) to refine your analysis is more in line with your findings and your research framework in general. However, while this approach is more suitable, it still relies on a ratio-based sample split. Beyond the age of the firm, you have not provided any non-ratio descriptive statistics for the firms in your sample. Is there a specific reason for this omission? Providing such descriptives would offer readers more context on the firms analyzed and strengthen the validity of your conclusions, particularly regarding SMEs.

**Our responses**: Thank you for your valuable comment. We focused on ratio-based variables to ensure unit consistency with our dependent variable (Z-score), which is a standardized measure. Including non-ratio descriptives could complicate interpretation due to scale differences.

**Reviewer #3**: The paper provides important insights about the association between financial constraints and corporate bankruptcy risks. However, some areas still need improvements.
- For the third hypothesis, author(s) should elaborate more on the interactive effect of financial constraints and cash holding on bankruptcy risk as this issue represents a focal point in the current research.

**Our responses**: Thank you for your suggestion. We have revised the section to elaborate on how cash holdings can moderate the impact of financial constraints on bankruptcy risk. The revised section is highlighted on pages 10 and 11.

- In table 1 descriptive statistics it is noted that the mean of leverage ratio (the ratio of total debt to total assets) is 243.2 % which is out of logic. The same also for the median, max and min values. Please check and confirm the descriptive statistics for leverage ratio.

**Our responses**: Thank you for pointing out the issue with the leverage ratio. Upon review, we identified an error in the calculation of this variable. As a result, we have removed leverage from our analysis and updated the manuscript accordingly. We appreciate your careful attention to detail.

- The results of Table 8 that presents the robustness findings are not discussed in the “Discussion section” in the main text. Please refer to the results of table 8 in the discussion section.

**Our responses**: Thank you for the comment. We have now referred to the results of Table 8 in the Discussion section and briefly explained the consistency across both exchanges. As suggested, Table 8 has been moved to Appendix B.

- In page 23 author(s) indicate that the results of table 5 show that leverage positively impact the probability of default risk. However, the results in table 5 do not show positive impact, but rather indicate that increases in leverage decrease distress risk.

**Our responses**: Thank you for your careful reading. Upon review, we found an error in the interpretation and the calculation of the leverage variable. Due to this issue, we have excluded leverage variable from our final model and updated the manuscript accordingly.
