# Supplementary material for: Financial constraints and corporate bankruptcy risks in China: The buffer role of cash holdings
Source: PLoS One. 2026 Jan 27;21(1):e0341114. doi: 10.1371/journal.pone.0341114 (PMC12843545; doi:10.1371/journal.pone.0341114)
Supplement: Attachment — Submitted filename: Response_to_Reviewer_auresp_3.docx [file pone.0341114.s005.docx]

PONE-D-24-39978R2

**FINANCIAL CONSTRAINTS AND CORPORATE BANKRUPTCY RISKS IN CHINA: THE BUFFER ROLE OF CASH HOLDINGS**

Review Comments to the Author

Reviewer #1: The authors have addressed my previous comments with reasonable improvements. Below, I provide my final remarks, following my earlier points.
1. Thank you for examining the differences between the exchanges. If you prefer, you may place Table 8 in the appendix, as no substantial conclusions can be drawn from it.

**Our response:** We sincerely appreciate the insightful suggestions. Following the reviewer's feedback, we have moved Table 8 to Appendix B in the previous manuscript.

2. I appreciate the authors’ rationale regarding the approach to the main results. While I would still prefer to see AWW included in Table 2, I acknowledge that this is not critical to the decision on the paper’s acceptance, so I will not insist on it.

**Our response:** Thank you for your valuable comment. We have decided to keep AWW included in Table 2.

3. Thank you for omitting the winsorization. This approach is more transparent. If you wish to do so, the original results could be given in an online appendix for reference.

**Our response:** Thank you for your thoughtful suggestion. At this stage, we prefer not to include the original results in an online appendix, as we believe the revised version better reflects the focus of our study.

4. Thank you for the clarification provided.

**Our response:** We sincerely thank anonymous reviewers.

5. The revised split appears to be more accurate, and I appreciate the decision to move away from the previous version.

**Our response:** We sincerely thank anonymous reviewers.

6. The conclusion now carries greater validity, as the SME classification is better justified. Nevertheless, **the classification of SME's is still not the most widely used one**, and **raw descriptive statistics are not provided**. I will not insist on these, but both the authors and editors should be aware of this limitation.

**Our response:** We sincerely thank the anonymous reviewer for this constructive comment. We acknowledge that the SME classification based on the Fama and French (1993) size breakpoint is not the most widely used approach, particularly in the context of China. In response, we have explicitly acknowledged this limitation in the conclusion. The revised section is highlighted on page 27.

Reviewer #3: Thank you for considering the comments. However, regarding the calculations **for leverage ratio (comment 3), authors indicate that they identified an error in the calculation of this variable (leverage ratio).** As a result, they have removed leverage from analysis and updated the manuscript accordingly. However, the variable Leverage is still included into the calculations of variables (Z_FC and AWW). Please confirm that the leverage ratio used in the calculations of the mentioned variables is right.

**Our response:** Thank you for your valuable comment. We confirm that the leverage ratio used in the calculations of AWW and Z_FC proxies has been correctly computed.
