# Supplementary material for: Financial constraints and corporate bankruptcy risks in China: The buffer role of cash holdings
Source: PLoS One. 2026 Jan 27;21(1):e0341114. doi: 10.1371/journal.pone.0341114 (PMC12843545; doi:10.1371/journal.pone.0341114)
Supplement: Attachment — Submitted filename: ResponseReport.docx [file pone.0341114.s006.docx]

PONE-D-24-39978R3

FINANCIAL CONSTRAINTS AND CORPORATE BANKRUPTCY RISKS IN CHINA: THE BUFFER ROLE OF CASH HOLDINGS

**While both reviewers recommended acceptance**, **I have carefully examined the paper as an editor and identified several major concerns that must be addressed before the manuscript can be considered further**. These revisions are essential to improve the theoretical rigor, methodological transparency, and overall scholarly contribution of the study.

Below, I outline the key points that require your attention. Please respond comprehensively to each issue in your revision and accompanying response letter.

1. The manuscript’s theoretical grounding is superficial and fragmented. The discussion of pecking order, trade-off, agency, and market-timing theories is descriptive rather than integrative. The link between these theories and the hypothesized relationships is weak, with no clear conceptual mechanism explaining how cash holdings buffer financial constraints.
Develop a unified conceptual framework showing the causal pathways linking financial constraints, cash holdings, and bankruptcy risk. Explicitly justify the buffer mechanism theoretically—e.g., through liquidity risk mitigation, precautionary saving behavior, or financing flexibility channels.

**Our response:** We have significantly revised the Literature Review to move beyond a descriptive list of theories. We now present a unified conceptual framework that integrates Market Timing, Trade-off, and Precautionary Savings theories. The revised Literature Review section is highlighted on pages 5-12.

2. Presenting each hypothesis with both positive and negative expected directions (e.g., H1A/H1B, H2A/H2B) is conceptually meaningless. It indicates the absence of a clear theoretical stance and weakens the research design.
Reformulate each hypothesis with a single, theory-driven directional expectation grounded in the literature. Remove the dual-sided hypothesis structure entirely.

**Our response:** We thank the reviewer for the critique regarding our hypothesis structure. We have completely removed the dual-sided structure (H1A/H1B) and reformulated our hypotheses to reflect a single, theory-driven stance. The revised Literature Review section is highlighted on pages 5-12.

3. The literature review is overly descriptive and outdated, relying heavily on older studies and lacking synthesis of recent (post-2020) works on liquidity buffers, macroprudential regulation, and bankruptcy prediction in China.
Condense repetitive sections, integrate recent literature (2021–2024), and structure the review thematically (theory → empirical contradictions → research gap). Emphasize what distinguishes the Chinese institutional setting and how this context strengthens your contribution.

**Our response:** We appreciate the reviewer’s guidance on modernizing our literature review. We have significantly condensed repetitive theoretical descriptions and restructured the review thematically with new references.  The revised Literature Review section is highlighted on pages 5-12.

4. The Z_FC and AWW indices are used without verifying their suitability for the Chinese context. The manuscript also claims to have removed Leverage due to a calculation error but still retains it in both indices, causing inconsistency.
Validate these indices empirically (e.g., via correlation or PCA tests) to confirm their relevance for Chinese firms. Ensure internal consistency by either recalculating the indices without Leverage or clearly justifying its inclusion.

**Our response:** We appreciate the editor’s comments regarding the suitability of the Z_FC and AWW indices and the inconsistency involving Leverage. To address these fundamental concerns, we have entirely replaced the previous indices with the SA Index (Hadlock & Pierce, 2010) as the sole proxy for financial constraints in the revised manuscript. We selected the SA Index because it relies exclusively on firm size and age, which are relatively exogenous variables, thereby avoiding the endogeneity problems inherent in leverage-based measures like the Z_FC or AWW. The revised section is highlighted on page 13.

Consequently, this substitution resolves the inconsistency issue, as Leverage is no longer included in the constraint construction nor the regression model.

5. The justification for employing dynamic system GMM is inadequate. The inclusion of the lagged Z-score is not theoretically motivated, and the use of 78 instruments risks overfitting. Additionally, the manuscript reports the J-statistic instead of the Hansen test, which is more robust for over-identification.
Explain why the dynamic specification is required (e.g., persistence in financial distress). Limit the number of instruments (ideally fewer than the number of groups) to avoid weak identification. Replace the J-statistic with the Hansen test and report AR(1) and AR(2) diagnostics explicitly. Discuss instrument validity in the text.

**Our response:** We appreciate the editor’s valuable feedback on the econometric specification. In the revised manuscript, we have strengthened the justification for the GMM estimation, arguing that bankruptcy risk exhibits persistence; specifically, past financial distress heavily conditions current risk due to reputation effects and long-term debt obligations, making the inclusion of the lagged dependent variable theoretically essential.

To address the concern of overfitting, we implemented the collapse option and restricted lag depths, ensuring the instrument count (45) remains strictly below the number of firms (groups), thereby improving the power of the overidentification test.

Furthermore, we have replaced the generic J-statistic with the Hansen test, and we explicitly report the AR(1) and AR(2) diagnostics. The insignificant Hansen and AR(2) results confirm the validity of our instruments and the absence of second-order serial correlation.

6. The fixed effects are inconsistently applied across models. FEM includes industry and year dummies, but the GMM section does not specify whether these are retained.
Apply a consistent fixed-effects structure across all models (industry and year) or clearly justify any deviation.

**Our response:** We sincerely thank the editor for pointing out the inconsistency in the fixed effects structure and acknowledge this oversight in the previous version. In the revised manuscript, we have excluded year and industry fixed effects from the fixed-effects estimation in the revised manuscript to align with the specific econometric properties of each estimator. However, for the Two-step system GMM estimation, we use both industry and year fixed effects (i.industry and i.year) in the instrument matrix. This deviation is justified because the Two-step system GMM allows us to control for sector-specific heterogeneity and temporal macroeconomic shocks rigorously.

7. The manuscript identifies endogenous variables but does not explain the instrument selection strategy or lag structure. Robustness checks are limited to alternative proxies and firm-size subsamples, omitting alternative distress measures.
Describe the choice and lag depth of instruments in detail. Add at least one alternative bankruptcy risk measure (e.g., O-score, Altman 1993 revision, or distance-to-default) to enhance robustness.

**Our response:** We sincerely thank the editor for this meticulous and constructive comment. We have substantially revised the methodology to detail our instrument selection strategy transparently, specifying that we treat the lagged dependent variable and key financial regressors as endogenous, instrumented using their own specific lag depths (strictly restricted to lags 2-2, and lags 3-3 for the interaction term) to address simultaneity while using the 'collapse' option to prevent instrument proliferation. Strictly exogenous variables (e.g., FAT, industry, and year effects) are included in the standard IV matrix. Furthermore, to address the concern regarding limited robustness, we have expanded our analysis by re-estimating the model using three alternative bankruptcy risk measures: the ZM-score, Z'-score, and Z"-score; the consistency of these results with our baseline findings significantly strengthens the validity of our conclusions regarding liquidity–distress dynamics.

8. The discussion primarily repeats coefficient signs without exploring their economic meaning. The interaction term (Z_FC × CASH) lacks visual or quantitative illustration.
Include marginal-effect or interaction plots to demonstrate moderation effects. Discuss economic significance and managerial relevance rather than only statistical results.

**Our response:** We appreciate the editor’s constructive suggestion to deepen the interpretation of the interaction results. Following your feedback, we have introduced Table 6 and a marginal-effect plot (Figure 1) that visually demonstrates how cash holdings moderate the impact of financial constraints on bankruptcy risk across different liquidity zones. Furthermore, we have thoroughly revised the discussion to prioritize economic significance and managerial relevance, explicitly interpreting the reversal in marginal effects as evidence of cash serving as a strategic stability buffer and a decoupling mechanism, rather than merely reporting statistical coefficient signs. The revised section is highlighted on pages 19-20.

9. Heteroskedasticity and serial correlation are noted but not addressed in sufficient detail.
Discuss the implications of these diagnostic results. If necessary, rerun GMM with collapsed instruments or shorter lags to improve test performance. Specify whether standard errors are robust or clustered and justify this choice.

**Our response:** We thank the editor for the technical scrutiny regarding the GMM specification. We confirm that our estimation strategy explicitly addresses these diagnostic concerns through the specific options employed in the xtabond2 command.

As detailed in our revised methodology, we employ the Two-step System GMM estimator with the robust option. In the context of xtabond2, this automatically reports standard errors that are robust to heteroskedasticity and clustered at the firm level. Crucially, this option applies the Windmeijer (2005) finite-sample correction to the standard errors, ensuring that the test statistics are reliable and not biased downwards, which is a common issue in two-step estimation.

Furthermore, we utilized the collapse option and strictly limited lag depths (specifically lag(2 2) for main variables and lag(3 3) for the interaction term) to prevent instrument proliferation and preserve the power of the Hansen test.

We report the AR(1) and AR(2) test results in the "Empirical Results" section, interpreting the significant AR(1) and insignificant AR(2) as evidence validating the absence of serial correlation in the error terms.

10. The paper contains redundant sentences, grammatical issues, and inconsistent variable names.
Undertake a professional English edit, eliminate redundancy, and ensure consistent names. Revise the abstract to emphasize motivation, methods, main results, and contribution succinctly.

**Our response:** We sincerely appreciate the feedback on presentation quality and have undertaken professional proofreading to correct errors, remove redundancy, and ensure variable consistency throughout the manuscript. Additionally, we have completely revised the Abstract to succinctly highlight the study's motivation, methodology, main results, and contributions as requested.

11. The conclusion reiterates findings without reflecting on their implications. Theoretical and policy contributions are generic, and limitations are not tied to future research directions.
Reframe the conclusion to emphasize (a) theoretical advancement in understanding liquidity–distress dynamics, (b) managerial and policy relevance for emerging markets, and (c) clear directions for future comparative or institutional research.

**Our responses:** We sincerely appreciate the editor’s insightful and constructive feedback regarding the Conclusion section. We acknowledge that the previous version was primarily descriptive and lacked a deep discussion of implications. Following your guidance, we have completely rewritten the Conclusion to explicitly highlight the theoretical advancements in liquidity–distress dynamics, discuss specific managerial and policy implications for emerging markets like China, and link our limitations to meaningful avenues for future institutional research. The revised section is highlighted on pages 29-32.

**References**

- Hadlock CJ, Pierce JR. New Evidence on Measuring Financial Constraints: Moving Beyond the KZ Index. Rev Financ Stud. 2010;23: 1909–1940. doi:10.1093/rfs/hhq009
- Windmeijer F. A finite sample correction for the variance of linear efficient two-step GMM estimators. Journal of Econometrics. 2005;126: 25–51. doi:10.1016/j.jeconom.2004.02.005
